# Association of Serum Zinc Status with 5-Year Clinical Outcomes in Women with Breast Cancer and Type 2 Diabetes: A Retrospective Cohort Study Using TriNetX

**DOI:** 10.3390/healthcare14091130

**Published:** 2026-04-23

**Authors:** Jui-Kun Chiang, Po-Chen Chiang, Malcolm Koo

**Affiliations:** 1 Department of Family Medicine, Dalin Tzu Chi Hospital, Buddhist Tzu Chi Medical Foundation, Chiayi 622401, Taiwan; 2 School of Medicine, College of Medicine, National Taiwan University, Taipei 10051, Taiwan; 3 Department of Medical Research, Dalin Tzu Chi Hospital, Buddhist Tzu Chi Medical Foundation, Chiayi 622401, Taiwan; 4 Dalla Lana School of Public Health, University of Toronto, Toronto, ON M5T 3M7, Canada

**Keywords:** zinc, breast cancer, diabetes, survival, intensive care unit, hospitalization

## Abstract

**Background/Objectives**: Zinc deficiency has been associated with increased cancer-related mortality, yet its prognostic relevance in patients with breast cancer and comorbid diabetes remains unclear. This study evaluated the association between zinc deficiency and adverse 5-year clinical outcomes in this population. **Methods**: This retrospective cohort study used the TriNetX database to identify women aged ≥20 years with breast cancer and type 2 diabetes who had recorded serum zinc levels within 3 months before the index date during the period from 1 January 2013 to 4 April 2026. Patients were categorized into zinc-deficiency, normal-zinc, or high-zinc groups. Outcomes included all-cause mortality, hospitalization, intensive care unit admission, and emergency department visits at the 1-, 3-, and 5-year follow-ups. Cox proportional hazards regression models were applied after 1:1 propensity score matching across 12 demographic and clinical variables. **Results**: Among 648 eligible patients, 282 had zinc deficiency, 311 had normal zinc levels, and 55 had high zinc levels. After matching, 218 remained in each of the zinc-deficient and control groups. Zinc deficiency was associated with higher all-cause mortality at 1 year (hazard ratio [HR], 2.45; 95% CI, 1.41, 4.28), 3 years (hazard ratio [HR], 2.09; 95% CI, 1.34, 3.28), and 5 years (HR, 1.92; 95% CI, 1.27, 2.92), as well as increased risks of emergency department visits, hospitalization, and ICU admission across follow-up periods. No significant differences were observed between the high-zinc and zinc-normal groups, possibly due to limited sample size. Subgroup analyses identified several exploratory subgroup-specific associations. **Conclusions**: Zinc deficiency was associated with poorer clinical outcomes in women with breast cancer and type 2 diabetes. However, because low serum zinc may also reflect malnutrition, systemic inflammation, frailty, or disease burden, these findings should be interpreted as hypothesis-generating rather than causal.

## 1. Introduction

Breast cancer is one of the most common cancers worldwide. According to the 2022 GLOBOCAN estimates, female breast cancer accounted for approximately 2.31 million new cases and 665,684 deaths globally, ranking as the most frequently diagnosed cancer among women [1]. Five-year survival for early-stage breast cancer now exceeds 90% [2], reflecting advances in screening, early diagnosis, and multimodal treatment, including surgery, chemotherapy, radiotherapy, and targeted therapies.

Globally, the burden of diabetes continues to rise. The International Diabetes Federation Diabetes Atlas (2025) reports that 11.1% of adults aged 20–79 years are living with diabetes [3]. Approximately 8–18% of patients with cancer also have diabetes, and several cancer therapies may increase the risk of developing diabetes mellitus [4]. Diabetes can influence treatment selection and may adversely affect cancer prognosis. Recent studies have also shown that diabetes is an independent risk factor for breast cancer development [5]. In addition, patients with diabetes may experience different treatment patterns and clinical outcomes compared with their nondiabetic counterparts. Although the underlying pathophysiological links between diabetes and breast cancer remain incompletely understood, proposed mechanisms include hyperinsulinemia, insulin resistance, and insulin-like growth factor signaling pathways [6,7,8], which may contribute to tumor cell proliferation, survival, and progression.

Zinc is an essential trace element involved in numerous biological processes. Approximately 85% of total body zinc is stored in skeletal muscle and bone [9], and zinc acts as a chemical messenger in insulin synthesis, storage, and secretion [10]. Globally, zinc deficiency (ZD) affects more than two billion people [11] and is associated with impaired appetite, poor wound healing, hair loss, and increased oxidative stress [12]. It has also been linked with cardiometabolic disorders, including cardiovascular disease, hypertension, dyslipidemia, and type 2 diabetes [13]. Systematic reviews have reported potential benefits of zinc supplementation in improving glucose control and insulin resistance in pre-diabetes and type 2 diabetes [14,15].

Low zinc levels have also been associated with higher mortality in several studies of patients with breast cancer [16,17]. In addition to its role in systemic metabolism, zinc may also be relevant to breast cancer progression through its effects on cellular signaling and tumor behavior. Experimental evidence suggests that hyperglycemic conditions can enhance the motile activity of breast cancer cells and increase the expression of zinc transporters such as ZIP6 and ZIP10, supporting a possible role of zinc transport in tumor behavior under high-glucose conditions [18]. More broadly, intracellular zinc homeostasis is regulated by two major transporter families, ZIP and ZnT, which mediate zinc influx and efflux across cellular membranes and thereby influence zinc-dependent signaling pathways. Several zinc transporters, particularly ZIP6, ZIP7, and ZIP10, have been associated with breast cancer progression and may contribute to tumor growth, motility, invasion, metastasis, and treatment resistance [19]. Some studies have also shown that patients with breast cancer who had a high serum copper-to-zinc ratio had an increased mortality risk, suggesting that lower zinc levels may be predictive of poorer outcomes [20,21]. However, not all studies have reported consistent associations between zinc levels and survival after breast cancer diagnosis [22]. Despite these findings, limited research has examined how zinc status influences outcomes specifically in patients with both diabetes and breast cancer in clinical settings.

Further mechanistic evidence provides additional biological rationale for investigating zinc in this population. Dysregulated zinc transport may alter intracellular zinc distribution and potentially contribute to oxidative stress, impaired DNA repair, immune dysfunction, and pro-tumorigenic signaling pathways relevant to breast cancer progression [23]. Complementary evidence suggests that zinc deficiency may alter the expression of microRNAs involved in oxidative stress, inflammatory regulation, and cancer-related signaling pathways, including miR-21 and miR-31 [24,25]. Because the roles of these microRNAs are context-dependent, and miR-144/miR-144-3p may exert tumor-suppressive effects in some cancer settings [26], these findings should be interpreted as indicating possible zinc-related microRNA dysregulation rather than a single uniform oncogenic mechanism. Together, these observations provide a plausible biological rationale for examining serum zinc status in this population, but the underlying mechanistic pathways remain incompletely understood and were not directly evaluated in the present study. In addition, the relationship between serum zinc status and clinical outcomes in patients with breast cancer and diabetes is likely multifactorial. Lower serum zinc levels may reflect not only altered zinc biology but also broader clinical vulnerability, including general nutritional status [27], systemic inflammation [28], and treatment-related effects [29], all of which may confound the association between zinc status and prognosis. Despite these considerations, limited research has examined the association between serum zinc status and clinical outcomes specifically in women with both breast cancer and type 2 diabetes. This is a clinically relevant gap because serum zinc in this population may reflect multiple interrelated processes, including metabolic dysfunction, nutritional status, systemic inflammation, and overall disease burden. We therefore examined whether lower serum zinc levels were associated with increased risks of mortality and adverse clinical outcomes in women with breast cancer and type 2 diabetes. However, because serum zinc may also reflect disease burden, nutritional status, systemic inflammation, treatment-related effects, or broader clinical frailty, reverse causation cannot be excluded.

## 2. Materials and Methods

### 2.1. Study Design and Data Source

This retrospective cohort study utilized the TriNetX platform (TriNetX LLC, Cambridge, MA, USA), an international collaborative health research network that provides access to real-time electronic health records. The query was conducted using the TriNetX Global Collaborative Network. The database contains de-identified patient data, including demographics, clinical diagnoses coded using International Classification of Diseases, 10th Revision, Clinical Modification (ICD-10-CM), medical procedures classified by ICD-10-PCS or Current Procedural Terminology, laboratory results mapped with Logical Observation Identifiers Names and Codes (LOINC), and healthcare utilization records [30].

As of April 2026, the TriNetX network included data from more than 150 healthcare organizations worldwide, representing over 250 million patients. These organizations were primarily academic medical centers and included mixed-care settings such as hospitals, affiliated institutions, and outpatient clinics. For the present analysis, data were contributed by 45 healthcare organizations within the TriNetX Global Collaborative Network. Data undergo standardized preprocessing to enhance consistency and reduce missingness. Although the network spans multiple geographic regions, detailed site-level information on participating countries and the geographic distribution of the analytic cohort was not available in the dataset used for this study.

Because TriNetX contains only anonymized records, written informed consent was not required. This study protocol was approved by Institutional Review Board of Dalin Tzu Chi Hospital, Buddhist Tzu Chi Medical Foundation (B11402057).

### 2.2. Patient Selection and Covariates

Women aged 20 years or older who had visited participating healthcare organizations between 1 January 2013 and 4 April 2026 were eligible for inclusion in the initial patient pool. Breast cancer was identified using ICD-10-CM code C50 recorded on at least three occasions. Because a breast cancer-specific external validation study for this threshold was not available in the TriNetX setting, this criterion should be regarded as a pragmatic operational definition intended to improve specificity and reduce potential false-positive classification in an electronic health record database [31]. The index date was defined as the date of the third recorded breast cancer diagnosis meeting this criterion and was used as the operational cohort-entry date for follow-up, rather than as a measure of the true onset of disease. In the second step, patients with breast cancer who also had a diagnosis of type 2 diabetes (ICD-10-CM: E11) within one year before the index date were identified. This time window was intended to capture active type 2 diabetes status near cohort entry rather than remote historical diagnoses. Because type 2 diabetes is generally a chronic condition requiring ongoing follow-up, this criterion likely captured many patients receiving active diabetes care; however, some patients with longstanding diabetes may still have been missed if the diagnosis was not recoded during that period. Eligible patients were further required to have a recorded serum zinc measurement within 3 months before the index date. Patients with type 1 diabetes and those without available serum zinc data were excluded.

Final cohort construction was performed using 1:1 propensity score matching. Fourteen candidate variables were initially evaluated, including demographic factors (age at index, sex, and race or ethnicity); comorbidities (essential hypertension, cerebral infarction, malnutrition, hyperlipidemia, and chronic kidney disease); breast cancer treatment and procedure variables (mastectomy, radiotherapy, and chemotherapy); and laboratory and medication-related variables (hemoglobin A1c, albumin, and tamoxifen use). Because mastectomy, radiotherapy, and chemotherapy had insufficient data for reliable matching, they were excluded from the final model. In the final propensity score matching analysis, race was represented by two indicator variables (White and Black or African American), and both hemoglobin A1c and albumin were entered in continuous and dichotomized forms. Accordingly, the final matching model included 12 covariate terms: age, White race, Black or African American race, hypertension, stroke, dyslipidemia, chronic kidney disease, malnutrition, mean hemoglobin A1c, hemoglobin A1c > 7%, mean albumin, and albumin < 3.5 g/dL. Detailed reproducibility information, including study variable definitions, cohort-building decisions, and the specific ICD-10-CM, CPT, and LOINC codes used in the TriNetX query, is provided in Supplementary Table S1.

Patients were categorized into three groups according to sex-specific reference considerations for low serum zinc status and prior clinical studies using similar operational thresholds [32,33,34]. Because the present cohort consisted entirely of women, serum zinc concentrations below 70 μg/dL were used to define inadequate zinc status on the basis of published reference considerations [32,33]. The remaining categories were defined a priori to distinguish non-deficient and elevated zinc groups, consistent with prior observational studies using similar serum zinc classifications [34,35]. Accordingly, zinc deficiency (ZD) was defined as <70 μg/dL, the zinc-normal control (ZC) group as 70–120 μg/dL, and the high-zinc (ZH) group as >120 μg/dL. All groups were followed for up to five years or until the date of data analysis on 4 April 2026. TriNetX does not apply statistical imputation to missing values. All analyses were conducted using complete data.

### 2.3. Outcome Measurement

For all-cause mortality, death was treated as the event of interest. For non-fatal outcomes, patients were followed from the index date until the occurrence of the outcome, the end of the corresponding follow-up window, or the date of last available record in the database (4 April 2026), whichever occurred first.

### 2.4. Statistical Analysis

Data were collected and analyzed on 4 April 2026, using the TriNetX Analytics platform. Baseline characteristics of the groups were reported as means (standard deviations), and counts (percentages), as appropriate. To address imbalances in baseline covariates, 1:1 propensity score matching was performed using the 12 variables listed above. Matching was completed using a greedy nearest-neighbor algorithm with a caliper width of 0.1 pooled standard deviations, consistent with the matching procedure implemented in the TriNetX platform. In the platform interface, this caliper is predefined rather than user-specified. This caliper restricts matched pairs to patients with very similar propensity scores and represents a relatively narrow matching tolerance. Covariate balance before and after propensity score matching was assessed numerically using standardized mean differences (SMDs) and displayed graphically using a love plot. An SMD < 0.1 was considered to represent acceptable balance [36].

After matching, event risk for each outcome was estimated using Cox proportional hazards regression models, with results presented as hazard ratios (HRs) and 95% confidence intervals (CIs). Proportional hazards assumptions were evaluated using the Grambsch and Therneau test [37] based on scaled Schoenfeld residuals, as implemented in the TriNetX platform [38]. As a sensitivity analysis to assess the robustness of the observed associations to potential unmeasured confounding, E-values were calculated for the hazard ratio estimates and their corresponding lower confidence limits. Kaplan–Meier curves were generated to compare event-free distributions between groups, with statistical significance assessed using the log-rank test. Survival probabilities were obtained from the TriNetX platform as aggregated, de-identified time-to-event estimates and exported as discrete time-point survival probabilities. These exported values were then used to plot the Kaplan–Meier curves in R statistical software (version 4.5.3, R Foundation for Statistical Computing, Vienna, Austria). Although patient-level data were not accessible outside the TriNetX platform, the figure-generation process is reproducible using the same exported survival probabilities and the same plotting steps in R. A *p*-value < 0.05 was considered statistically significant. Subgroup analyses were conducted to assess whether results varied by age, hypertension, dyslipidemia, chronic kidney disease, breast cancer treatments, hemoglobin A1c (HbA1c), and albumin.

## 3. Results

**Figure 1:**
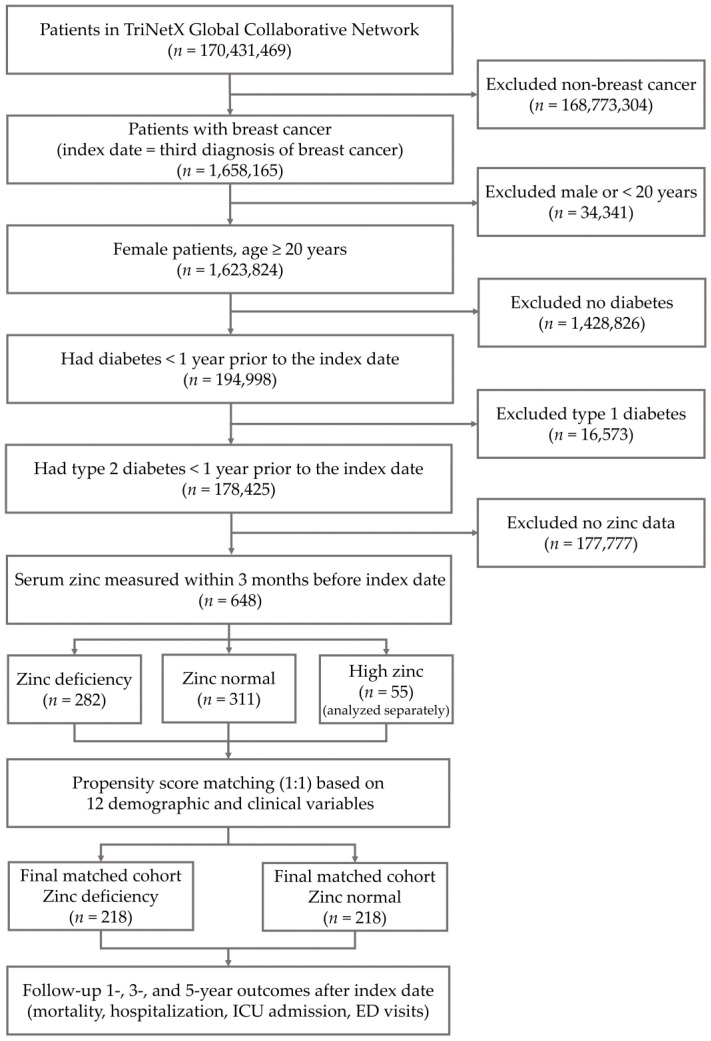
Flowchart of patient selection and propensity score matching.

A total of 648 female patients met the inclusion criteria, including 282 in the zinc deficiency (ZD) group, 311 in the zinc normal control (ZC) group, and 55 in the high-zinc (ZH) group. As shown in Figure 1, among 194,998 female patients aged 20 years or older with breast cancer who had diabetes within 1 year before the index date, 16,573 with type 1 diabetes and 177,777 without a serum zinc measurement within 3 months before the index date were excluded, leaving 648 patients with type 2 diabetes for analysis. After 1:1 propensity score matching, 218 patients remained in each of the ZD and ZC groups. The large number of excluded patients, most of whom lacked an available serum zinc measurement within the prespecified window, reflects the non-routine nature of zinc testing in usual care and may have contributed to selection bias.

Female patients aged 20 years or older with breast cancer were identified from the TriNetX Global Collaborative Network, with the index date defined as the third recorded breast cancer diagnosis. Among these, 194,998 had diabetes within 1 year before the index date. After excluding 16,573 patients with type 1 diabetes and 177,777 without serum zinc measurements within 3 months before the index date, 648 patients with type 2 diabetes remained for analysis. These were classified as zinc deficiency (*n* = 282), zinc normal (*n* = 311), or high zinc (*n* = 55), with the high-zinc group analyzed separately. A 1:1 propensity score matching analysis based on 12 demographic and clinical variables was performed for the zinc deficiency and zinc normal groups, yielding 218 patients in each matched group. Outcomes were assessed at 1, 3, and 5 years after the index date.

**Figure 2:**
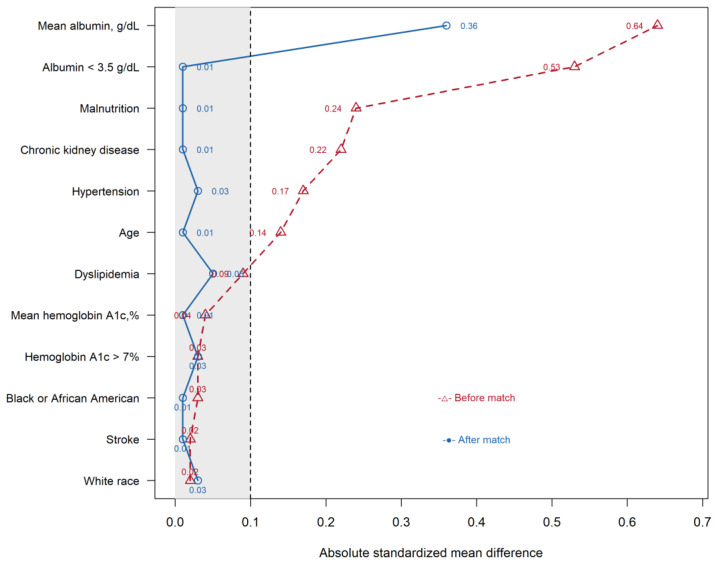
Love plot of absolute standardized mean differences (SMDs) before and after matching. The vertical dashed line at an absolute standardized mean difference (SMD) of 0.1 indicates the conventional threshold for acceptable covariate balance. Values to the left of this line suggest negligible imbalance between groups, whereas values to the right indicate meaningful residual imbalance. The shaded grey region (SMD < 0.1) represents the range in which covariates are considered well balanced after matching.

Table 1 presents the baseline characteristics of the ZD and ZC groups before and after propensity score matching. Before matching, several covariates showed imbalance between groups, particularly mean albumin, albumin < 3.5 g/dL, malnutrition, chronic kidney disease, and hypertension. After matching, balance improved for most covariates. Residual imbalance remained for continuous mean albumin (SMD = 0.360), whereas the dichotomized albumin variable was well balanced (SMD = 0.009). Baseline covariate balance before and after matching is shown in Figure 2.

**Figure 3:**
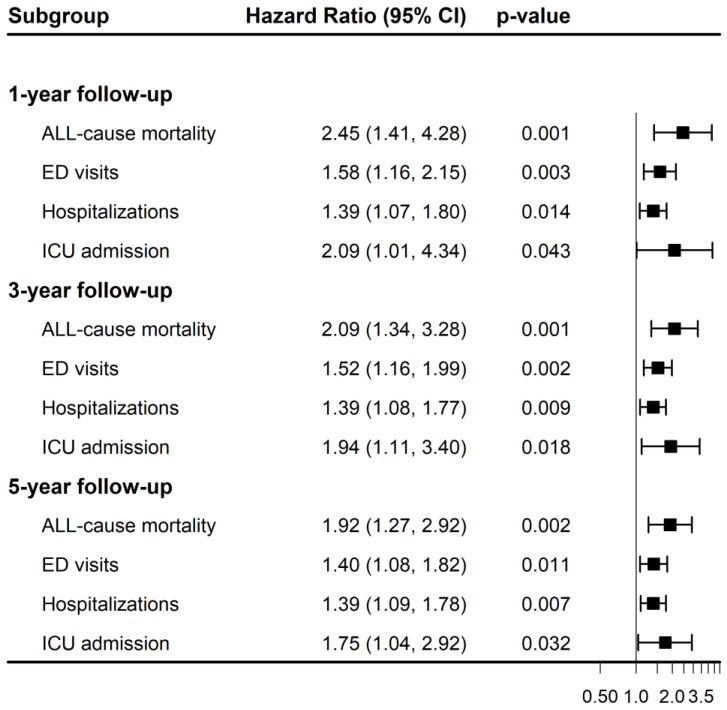
Forest plot displaying hazard ratios and 95% confidence intervals for primary outcomes at 1-, 3-, and 5-year follow-up intervals. CI, confidence interval; ED, emergency department; ICU, intensive care unit.

Kaplan–Meier event-free curves for the four outcomes at 1-, 3-, and 5-year follow-up are shown in Supplementary Figures S1–S3. The corresponding hazard ratios and 95% confidence intervals are summarized in Table 2 and Figure 3. After matching, the ZD group had a higher risk of all-cause mortality at 1 year (40 vs. 18 events; HR, 2.45; 95% CI, 1.41 to 4.28; *p* = 0.001), 3 years (53 vs. 30 events; HR, 2.09; 95% CI, 1.34 to 3.28; *p* = 0.001), and 5 years (57 vs. 36 events; HR, 1.92; 95% CI, 1.27 to 2.92; *p* = 0.002). The ZD group also had higher risks of emergency department visits at 1 year (97 vs. 72 events; HR, 1.58; 95% CI, 1.16 to 2.15; *p* = 0.003), 3 years (116 vs. 97 events; HR, 1.52; 95% CI, 1.16 to 1.99; *p* = 0.002), and 5 years (120 vs. 110 events; HR, 1.40; 95% CI, 1.08 to 1.82; *p* = 0.011); hospitalizations at 1 year (121 vs. 103 events; HR, 1.39; 95% CI, 1.07 to 1.80; *p* = 0.014), 3 years (136 vs. 123 events; HR, 1.39; 95% CI, 1.08 to 1.77; *p* = 0.009), and 5 years (138 vs. 125 events; HR, 1.39; 95% CI, 1.09 to 1.78; *p* = 0.007); and ICU admissions at 1 year (21 vs. 11 events; HR, 2.09; 95% CI, 1.01 to 4.34; *p* = 0.043), 3 years (32 vs. 20 events; HR, 1.94; 95% CI, 1.11 to 3.40; *p* = 0.018), and 5 years (35 vs. 25 events; HR, 1.75; 95% CI, 1.04 to 2.92; *p* = 0.032). To assess the robustness of the observed associations to potential unmeasured confounding, E-values were calculated for the hazard ratio estimates and their lower confidence limits, as shown in Table 2. All Schoenfeld residual test *p* values were greater than 0.05, suggesting no violation of the proportional hazards assumption in the reported models.

Although 55 patients were initially classified in the ZH group, only 46 could be retained after separate 1:1 propensity score matching with the ZC group. Given the small sample size of the ZH group, its results are presented separately in Table 3. No statistically significant differences were observed between the ZH and ZC groups for estimable outcomes at 1, 3, or 5 years. Specifically, the hazard ratios were 1.73 (95% CI, 0.86 to 3.45; *p* = 0.117) for 1-year emergency department visits, 1.81 (95% CI, 0.98 to 3.32; *p* = 0.053) for 1-year hospitalization, 1.38 (95% CI, 0.31 to 6.18; *p* = 0.670) for 3-year all-cause mortality, 1.58 (95% CI, 0.87 to 2.89; p = 0.130) for 3-year emergency department visits, 1.28 (95% CI, 0.74 to 2.19; *p* = 0.377) for 3-year hospitalization, 1.60 (95% CI, 0.90 to 2.85; *p* = 0.106) for 5-year emergency department visits, and 1.28 (95% CI, 0.74 to 2.19; *p* = 0.377) for 5-year hospitalization. Several outcomes were not estimable because of low event counts.

Exploratory subgroup analyses at the 1-, 3-, and 5-year follow-ups identified several subgroup-specific associations between zinc deficiency and adverse outcomes (Table 4, Table 5 and Table 6). At the 1-year follow-up, significant associations were observed for all-cause mortality among patients who had received antineoplastic chemotherapy and among those with albumin < 3.5 g/dL; for emergency department visits among patients aged ≥65 years, those with dyslipidemia, prior mastectomy, antineoplastic chemotherapy, and HbA1c > 7%; and for hospitalization among patients with hypertension, dyslipidemia, chronic kidney disease, antineoplastic chemotherapy, and HbA1c > 7%. At the 3-year follow-up, significant associations were observed for all-cause mortality among patients who had received antineoplastic chemotherapy; for emergency department visits among patients aged ≥65 years, those with dyslipidemia, those receiving antineoplastic chemotherapy, and those with HbA1c > 7%; for hospitalization among patients aged ≥65 years, those with dyslipidemia, chronic kidney disease, antineoplastic chemotherapy, and HbA1c > 7%; and for ICU admission among patients with dyslipidemia. At the 5-year follow-up, significant associations were observed for all-cause mortality among patients receiving antineoplastic chemotherapy; for emergency department visits among patients aged ≥65 years, those with dyslipidemia, and those with HbA1c > 7%; for hospitalization among patients aged ≥65 years, those with dyslipidemia, chronic kidney disease, antineoplastic chemotherapy, and HbA1c > 7%; and for ICU admission among patients with hypertension and dyslipidemia.

## 4. Discussion

### 4.1. Overall Interpretation

In this matched cohort of women with breast cancer and type 2 diabetes, zinc deficiency was associated with a higher risk of all-cause mortality at 1-, 3-, and 5-year follow-up, as well as higher risks of emergency department visits, hospitalization, and intensive care unit admission across all follow-up periods. The broadly consistent direction of the hazard ratios across multiple outcomes may also be compatible with residual confounding by broader clinical vulnerability, disease severity, or nutritional and inflammatory status. Zinc is an essential trace element involved in immune regulation, inflammatory control, and antioxidant defense [39]. The 5-year survival rate for all combined breast cancer stages is approximately 91% [40]. A long-term study reported that the 10-year overall survival was 58.3% for women in the highest quartile and 82.1% for those in the lowest quartile of the serum copper-to-zinc ratio [20]. A higher copper-to-zinc ratio, potentially reflecting lower zinc levels, was associated with poorer survival. Disruption of copper and zinc metabolism has been proposed as one possible contributor to carcinogenesis [41]. At the same time, prior evidence on zinc-related markers and breast cancer outcomes has been mixed rather than uniform. A prospective cohort study including 17,035 women found no overall association of dietary or serum zinc levels with recurrence-free, breast cancer-specific, or overall survival, although subgroup analyses suggested that intermediate or high zinc intake in the context of high phosphorus intake was associated with more favorable survival [16]. These inconsistencies suggest that any association between zinc-related measures and prognosis is unlikely to be uniform across all clinical settings and may be modified by population characteristics, nutritional background, or analytic approach.

Although the hazard ratio for all-cause mortality was highest at 1 year, significant associations remained evident at 3 and 5 years. This pattern may be consistent with both short-term clinical vulnerability and longer-term risk. However, these findings should be interpreted cautiously, because the observed associations may also reflect residual confounding, reverse causation, or other unmeasured factors. In particular, lower serum zinc levels may have reflected greater underlying illness burden, systemic inflammation, malnutrition, treatment-related effects, or broader clinical frailty rather than a temporally prior causal exposure. Some of the observed hazard ratio magnitudes, especially for all-cause mortality, may therefore have been influenced by bias or residual confounding. In this context, low serum zinc may be better understood as a marker of frailty and broader clinical vulnerability rather than as an isolated causal determinant of adverse outcomes. Accordingly, the biologic mechanisms discussed below are intended to provide clinical context for the observed associations rather than to support a causal interpretation.

### 4.2. Increased Infection Risk and Intensive Care Unit Admissions

Zinc is essential for immune competence and redox homeostasis. It serves as a catalytic or structural component of more than 300 enzymes and numerous transcription factors involved in DNA repair, cell proliferation, and inflammatory regulation [42,43]. Zinc also contributes to antioxidant defense through its role as a cofactor for superoxide dismutase and by regulating metallothioneins, which mitigate oxidative and inflammatory injury [44].

In patients with coexisting diabetes and breast cancer, these protective mechanisms may be particularly vulnerable. Zinc is required for insulin crystallization within pancreatic β-cells, a process mediated by the zinc transporter ZnT8 [45]. Reduced ZnT8 expression or function impairs insulin secretion and is associated with an increased risk of type 2 diabetes [46]. Chronic hyperglycemia further amplifies oxidative stress and activates NF-κB signaling, increasing cellular zinc demand to support antioxidant responses. Sustained demand may deplete circulating zinc levels, thereby weakening zinc-dependent antioxidant capacity and host defenses [47]. This biological context provides a plausible explanation for the increased risks of emergency department visits, hospitalization, and intensive care unit admission observed among zinc-deficient patients.

Subgroup findings suggest that these associations may be more evident in selected clinical contexts rather than uniformly across all patient groups. In the present analyses, patients with hypertension showed a higher risk of hospitalization at 1-year follow-up and a higher risk of intensive care unit admission at 5-year follow-up, but not a consistent increase in emergency department visits across time points. This more limited pattern is compatible with the view that zinc deficiency may interact with vascular comorbidity and reduced physiological reserve, while also indicating that subgroup-specific findings should be interpreted cautiously [48,49,50].

### 4.3. Impaired Immune Surveillance and Adverse Cancer Outcomes

Beyond acute illness, ZD may adversely influence long-term cancer outcomes through impaired immune surveillance and tumor-promoting inflammatory signaling. Zinc is integral to both innate and adaptive immunity, supporting natural killer cell function, cytokine regulation, and control of chronic inflammation. Deficiency states are associated with immune dysfunction, persistent inflammatory activation, and reduced capacity for tumor immune surveillance.

At the molecular level, dysregulated zinc metabolism has been linked to activation of pro-tumorigenic signaling pathways, including Wnt/β-catenin signaling, and to metabolic remodeling of the tumor microenvironment mediated by zinc-finger proteins such as ZEB1 [51,52]. Hypoxia-induced phase separation of zinc-finger proteins, including ZHX2, can further activate oncogenic transcriptional programs that promote tumor progression and metastasis [53]. Experimental and clinical studies have shown that zinc repletion enhances antioxidant defenses, reflected by increases in glutathione levels, total antioxidant capacity, and superoxide dismutase activity, counteracting oxidative stress commonly observed in diabetes and malignancy [54].

Zinc homeostasis also influences cancer cell survival. Zinc depletion may induce apoptosis or necrosis, whereas malignant cells can upregulate zinc importers to preserve intracellular zinc levels required for proliferation and survival [55]. Consistent with these mechanisms, recent multi-database analyses have identified zinc metabolism–related gene signatures, including ATP7B, BGLAP, and SLC39A11, that stratify breast cancer prognosis, with high-risk profiles associated with immune evasion and poorer survival [56]. Upregulation of zinc finger protein 207 has likewise been identified as a pan-cancer biomarker associated with aggressive tumor behavior and immune escape [57]. Together, these findings provide biological plausibility for the observed association between ZD and increased mortality in patients with breast cancer and diabetes.

### 4.4. Poor Metabolic Control in Diabetes

Zinc plays a central role in glucose metabolism and insulin signaling, providing a mechanistic link between zinc deficiency and adverse outcomes in patients with diabetes. Adequate zinc availability is required for insulin synthesis, crystallization, storage, and secretion within pancreatic β-cells, processes mediated in part by ZnT8 [45]. Impaired ZnT8 function disrupts insulin secretion and increases susceptibility to type 2 diabetes [46]. In peripheral tissues, zinc supports key insulin signaling pathways, including Akt2 and GSK-3β, which are essential for glucose uptake and metabolic homeostasis [58].

Zinc deficiency has been associated with insulin resistance, impaired glucose tolerance, and increased severity of diabetic complications. Persistent hyperglycemia may further intensify oxidative stress and cellular zinc demand, creating a self-reinforcing cycle in which metabolic dysregulation accelerates zinc depletion and worsens clinical outcomes. Consistent with this framework, our subgroup analyses showed that among patients with poor glycemic control, defined as hemoglobin A1c greater than 7%, zinc deficiency was associated with higher risks of emergency department visits and hospitalization at the 1-, 3-, and 5-year follow-ups, whereas no corresponding increase in all-cause mortality was observed. These findings suggest that poor glycemic control may modify the association between zinc deficiency and adverse outcomes primarily through greater clinical instability and acute care use rather than mortality. Glycemic status may therefore merit consideration as a stratification factor when evaluating the prognostic relevance of zinc deficiency in this population [38].

### 4.5. Subgroup Findings and Clinical Context

The subgroup analyses showed several exploratory patterns, although the direction and strength of associations differed by outcome and follow-up period. At the 3- and 5-year follow-ups, patients aged 65 years or older showed higher risks of emergency department visits and hospitalization in the zinc-deficient group than in the zinc-normal group. Patients with dyslipidemia showed a more consistent pattern, with higher risks of emergency department visits and hospitalization at the 1-, 3-, and 5-year follow-ups and higher risks of intensive care unit admission at the 3- and 5-year follow-ups. Among patients with chronic kidney disease, zinc deficiency was associated with higher risks of hospitalization across follow-up periods. Patients who had received antineoplastic chemotherapy also showed higher risks of multiple adverse outcomes, including all-cause mortality at the 1-, 3-, and 5-year follow-ups, together with higher risks of emergency department visits or hospitalization at several time points.

Among patients with hypertension, the pattern was more limited. Zinc deficiency was associated with a higher risk of hospitalization at the 1-year follow-up and with a higher risk of intensive care unit admission at the 5-year follow-up, but not with a consistent increase in emergency department visits across time points. This pattern may be consistent with prior evidence linking low serum zinc levels to adverse cardiovascular outcomes and increased hospitalization risk in populations with underlying vascular disease [48].

In contrast, prior mastectomy was not associated with a clear or persistent increase in adverse outcomes, apart from a higher risk of emergency department visits at the 1-year follow-up. These findings suggest that the clinical relevance of zinc deficiency may vary across patient subgroups and may be more evident in settings characterized by metabolic instability, vascular comorbidity, or more intensive cancer treatment exposure.

Overall, these subgroup findings should be interpreted cautiously. Because multiple subgroup analyses were performed without formal adjustment for multiple comparisons, the likelihood of false-positive findings is increased. In addition, some subgroup estimates were based on limited event numbers, which further reduces their statistical reliability. These analyses should therefore be regarded as exploratory and hypothesis-generating rather than confirmatory.

### 4.6. Clinical Implications

Collectively, susceptibility to acute illness and critical care use, impaired immune surveillance with cancer progression, and metabolic dysregulation in diabetes form a plausible clinical framework linking zinc deficiency to the adverse outcomes observed in this study.

From a clinical perspective, these findings suggest that serum zinc status may be relevant when evaluating women with breast cancer and coexisting type 2 diabetes, particularly in those with poorer glycemic control, dyslipidemia, chronic kidney disease, or exposure to antineoplastic chemotherapy, where several exploratory subgroup associations were observed. However, because this study was observational and the subgroup analyses were exploratory, the present findings do not support broad risk stratification or intervention solely on the basis of these subgroup patterns. Instead, they support further prospective evaluation of whether zinc assessment may help identify patients at higher risk of adverse outcomes in this population.

Importantly, the present findings do not support routine zinc supplementation beyond the correction of confirmed deficiency. Any correction strategy, including dietary modification or supplementation [59], should be interpreted cautiously and ideally evaluated in prospective studies before being incorporated into routine oncologic or metabolic care pathways.

### 4.7. Limitations

There were several limitations in the current study. First, owing to constraints of the TriNetX database, key tumor-related confounders, including cancer stage, tumor size, nodal involvement, tumor subtype, and performance status, were not available, and cause-specific mortality could not be determined. In addition, treatment-related variables such as chemotherapy and radiotherapy could not be included in the final propensity score model because of insufficient data completeness for reliable matching. As a result, the matched comparisons may still have been confounded by differences in underlying disease severity, biologic heterogeneity, and treatment intensity. Notably, residual post-matching imbalance remained for continuous albumin (SMD = 0.360), exceeding the conventional threshold for acceptable balance. As shown in both Table 1 and the love plot, covariate balance improved after matching for most variables, but residual imbalance persisted for continuous albumin. Because albumin is an important proxy for nutritional and inflammatory status, this residual imbalance suggests incomplete control of these factors and possible residual confounding. Accordingly, part of the observed associations may reflect differences in underlying clinical vulnerability rather than zinc status alone. Important variables such as dietary zinc intake or supplementation, socioeconomic status, and lifestyle factors were also not captured. Consequently, residual confounding cannot be fully excluded despite the use of propensity score matching, and the findings should be interpreted as associations rather than evidence of causality.

Second, the overall sample size of diabetic patients with breast cancer was modest, particularly after propensity score matching. This issue was especially relevant for the high-zinc group, which comprised only 46 patients after matching. The limited statistical power and wider confidence intervals around the effect estimates may have reduced our ability to detect meaningful differences and limited interpretability of the high-zinc analyses.

Third, the timing of serum zinc measurement relative to breast cancer diagnosis and treatment could not be standardized, which introduces temporal ambiguity. In addition, although fasting blood draws are commonly used in metabolic assessments, fasting status at the time of zinc measurement could not be verified. Because laboratory assays were obtained from multiple healthcare organizations within the TriNetX network, inter-institutional variability in zinc measurement cannot be fully excluded, and assay procedures could not be independently standardized within the analytic platform. Therefore, the potential influence of dietary intake, diurnal variation, fasting status, and laboratory heterogeneity on serum zinc levels cannot be excluded. More broadly, serum zinc should be interpreted in this study as an available circulating biomarker and an operational exposure measure rather than as a definitive indicator of baseline physiological zinc status. In patients with cancer, circulating zinc concentrations may also reflect acute illness, systemic inflammation, nutritional status, hypoalbuminemia, renal dysfunction, treatment-related metabolic changes, and overall disease burden. In addition, because zinc status was defined using a single available serum measurement within the pre-index window, longitudinal variability in zinc levels could not be assessed, and this measurement may not have represented longer-term zinc status over the course of disease and treatment.

Fourth, because TriNetX does not apply statistical imputation, the analysis was restricted to patients with available serum zinc measurements. This restriction may have introduced selection bias, as the included cohort may have differed systematically from patients without zinc data. In addition, detection bias cannot be excluded, because patients with greater clinical complexity, poorer overall health, or suspected nutritional or inflammatory disturbances may have been more likely to undergo serum zinc testing in routine practice.

Fifth, the study cohort was restricted to female patients. Given the rarity of male breast cancer and its distinct hormonal and biological characteristics, the findings are specific to female patients with breast cancer and diabetes and may not be generalizable to male patients. In addition, although TriNetX includes data from multiple institutions and regions, participating healthcare organizations may not fully represent other populations, clinical settings, or healthcare systems. Therefore, caution is warranted when generalizing these findings beyond the TriNetX network.

Finally, reverse causation should also be considered when interpreting the present findings. Although serum zinc was measured within 3 months before the index date, lower zinc levels may have reflected more advanced cancer burden, systemic inflammation, malnutrition, treatment-related effects, or greater overall clinical frailty rather than an independent causal contributor to adverse outcomes. In this context, zinc deficiency may function partly as a marker of poorer underlying health status and disease severity. Accordingly, the observed associations should not be interpreted as evidence of causality, and prospective studies with more clearly defined temporal sequencing are needed.

## 5. Conclusions

In this matched retrospective cohort of women with breast cancer and type 2 diabetes, zinc deficiency was associated with higher risks of all-cause mortality, emergency department visits, hospitalization, and intensive care unit admission across the 1-, 3-, and 5-year follow-up periods. Exploratory subgroup analyses suggested that some of these associations may be more evident in patients with dyslipidemia, chronic kidney disease, poor glycemic control, or antineoplastic chemotherapy exposure, although these subgroup findings should be interpreted cautiously. Overall, these findings suggest that zinc deficiency may serve as a marker of poorer prognosis and greater healthcare utilization in women with breast cancer and type 2 diabetes. However, given the observational design and the potential for residual confounding, reverse causation, and selection bias, the results should be interpreted as hypothesis-generating associations rather than evidence of a causal effect.

## Figures and Tables

**Table 1:** Baseline characteristics of patients before and after propensity score matching.

Variable	Before Matching			After Matching		
	ZD (*n* = 282)	ZC (*n* = 311)	SMD	ZD (*n* = 218)	ZC (*n* = 218)	SMD
Mean age at index, years (SD)	65.5 (12.1)	63.9 (11.1)	0.139	64.6 (12.0)	64.5 (10.9)	0.008
Race/ethnicity						
White	178 (63.1)	199 (64.0)	0.018	137 (62.8)	140 (64.2)	0.029
Black or African American	61 (21.6)	63 (20.3)	0.034	46 (21.1)	45 (20.6)	0.011
Comorbidities						
Hypertension	224 (79.4)	224 (72.0)	0.173	172 (78.9)	175 (80.3)	0.034
Stroke	10 (3.5)	10 (3.2)	0.018	10 (4.6)	10 (4.6)	<0.001
Dyslipidemia	160 (56.7)	163 (52.4)	0.087	119 (54.6)	124 (56.9)	0.046
Chronic kidney disease	79 (28.0)	58 (18.6)	0.223	49 (22.5)	49 (22.5)	<0.001
Malnutrition	58 (20.6)	37 (11.9)	0.237	34 (15.6)	35 (16.1)	0.013
Mean hemoglobin A1c, % (SD)	6.5 (1.5)	6.5 (1.2)	0.038	6.5 (1.5)	6.5 (1.3)	0.006
Hemoglobin A1c > 7%	81 (28.7)	93 (29.9)	0.026	66 (30.3)	69 (31.7)	0.030
Mean albumin, g/dL (SD)	3.5 (0.7)	3.9 (0.5)	0.644	3.5 (0.7)	3.8 (0.6)	0.360
Albumin < 3.5 g/dL	181 (64.2)	120 (38.6)	0.530	120 (55.0)	119 (54.6)	0.009

SMD, standardized mean difference; ZD, zinc deficiency; ZC, zinc normal control. Data are presented as number (percentage) unless otherwise indicated.

**Table 2:** Hazard ratios, E-values, and proportional hazards test results for clinical outcomes associated with zinc deficiency relative to the zinc-normal control group at 1-, 3-, and 5-year follow-up.

Outcome	Index Date to 1-Year Follow-Up				Index Date to 3-Year Follow-Up				Index Date to 5-Year Follow-Up			
	*n*		HR (95% CI)	Cox *p* [PH Test *p*]	*n*		HR (95% CI)	Cox *p* [PH Test *p*]	*n*		HR (95% CI)	Cox *p* [PH Test *p*]
	ZD	ZC	[E-Value (95% LCI)]		ZD	ZC	[E Value (95% LCI)]		ZD	ZC	[E-Value (95% LCI)]	
All-cause mortality	40	18	2.45 (1.41–4.28) [4.33 (2.17)]	0.001 (0.613)	53	30	2.09 (1.34–3.28) [3.60 (2.01)]	0.001 (0.386)	57	36	1.92 (1.27–2.92) [3.25 (1.86)]	0.002 (0.146)
ED visits	97	72	1.58 (1.16–2.15) [2.54 (1.59)]	0.003 (0.296)	116	97	1.52 (1.16–1.99) [2.41 (1.59)]	0.002 (0.533)	120	110	1.40 (1.08–1.82) [2.15 (1.37)]	0.011 (0.419)
Hospitalizations	121	103	1.39 (1.07–1.80) [2.13 (1.34)]	0.014 (0.981)	136	123	1.39 (1.08–1.77) [2.13 (1.37)]	0.009 (0.916)	138	125	1.39 (1.09–1.78) [2.13 (1.40)]	0.007 (0.986)
ICU admissions	21	11	2.09 (1.01–4.34) [3.60 (1.11)]	0.043 (0.807)	32	20	1.94 (1.11–3.40) [3.29 (1.46)]	0.018 (0.578)	35	25	1.75 (1.04–2.92) [2.90 (1.24)]	0.032 (0.184)

CI, confidence interval; ED, emergency department; HR, hazard ratio; ICU, intensive care unit; LCI, lower confidence interval; ZC, zinc normal control; ZD, zinc deficiency. Cox *p* indicates the *p* value from the Cox proportional hazards regression model, and PH test *p* indicates the *p* value from the Schoenfeld residuals test of proportionality. E-values were calculated for the point estimate and lower confidence limit to assess the minimum strength of association that an unmeasured confounder would need to have with both zinc deficiency and the outcome, conditional on the measured covariates, to explain away the observed association.

**Table 3:** Hazard ratios of clinical outcomes associated with the high-zinc group (*n* = 46) relative to the zinc normal control group (*n* = 46) after propensity score matching.

Duration of Follow-Up	Outcome	HR (95% CI)	Log Rank Test, *p*	Proportionality Test, *p*
Index date to 1-year follow-up				
	All-cause mortality	NE	NE	NE
	ED visits	1.73 (0.86–3.45)	0.117	0.648
	Hospitalizations	1.81 (0.98–3.32)	0.053	0.887
	ICU admissions	NE	NE	NE
Index date to 3-year follow-up				
	All-cause mortality	1.38 (0.31–6.18)	0.670	0.409
	ED visits	1.58 (0.87–2.89)	0.130	0.681
	Hospitalizations	1.28 (0.74–2.19)	0.377	0.075
	ICU admissions	NE	NE	NE
Index date to 5-year follow-up				
	All-cause mortality	NE	NE	NE
	ED visits	1.60 (0.9–2.85)	0.106	0.670
	Hospitalizations	1.28 (0.74–2.19)	0.377	0.377
	ICU admissions	NE	NE	NE

CI, confidence interval; ED, emergency department; HR, hazard ratio; ICU, intensive care unit; NE: not estimable due to low events.

**Table 4:** Subgroup analyses of adverse outcomes within one year of the index date in the zinc deficiency group versus zinc normal control group after propensity score matching.

Subgroup	All-Cause Mortality		ED Visits		Hospitalizations		ICU Admissions	
	HR (95% CI)	*p*	HR (95% CI)	*p*	HR (95% CI)	*p*	HR (95% CI)	*p*
Age, ≥65 years (143 pairs)	NE		1.81 (1.24–2.65)	0.002	1.34 (0.97–1.85)	0.072	NE	
Age, <65 years (65 pairs)	NE		1.42 (0.83–2.44)	0.196	1.31 (0.83–2.07)	0.244	NE	
Hypertension (194 pairs)	NE		1.04 (0.71–1.53)	0.823	1.44 (1.04–2.00)	0.029	NE	
Dyslipidemia (174 pairs)	1.62 (0.83–3.19)	0.157	1.60 (1.15–2.22)	0.005	1.46 (1.10–1.93)	0.008	NE	
Chronic kidney disease (84 pairs)	NE		1.41 (0.90–2.22)	0.133	1.67 (1.13–2.49)	0.009	NE	
Mastectomy (89 pairs)	NE		1.75 (1.09–2.79)	0.018	1.42 (0.93–2.16)	0.099	NE	
Antineoplastic chemotherapy (196 pairs)	2.17 (1.19–3.95)	0.010	1.55 (1.13–2.12)	0.006	1.42 (1.09–1.86)	0.009	1.25 (0.56–2.79)	0.586
Hemoglobin A1c, >7% (44 pairs)	NE		2.02 (1.05–3.88)	0.032	1.81 (1.05–3.12)	0.032	NE	
Albumin, <3.5 g/dL (74 pairs)	2.06 (1.05–4.05)	0.032	1.58 (0.96–2.59)	0.069	1.23 (0.79–1.89)	0.358	NE	

CI, confidence interval; ED, emergency department; HR: hazard ratio; ICU, intensive care unit; NE: not estimable due to low events.

**Table 5:** Subgroup analyses of adverse outcomes within three years of the index date in the zinc deficiency group versus zinc normal control group after propensity score matching.

Subgroup	All-Cause Mortality		ED Visits		Hospitalizations		ICU Admissions	
	HR (95% CI)	*p*	HR (95% CI)	*p*	HR (95% CI)	*p*	HR (95% CI)	*p*
Age, ≥65 years (143 pairs)	1.53 (0.87–2.69)	0.141	1.64 (1.19–2.27)	0.002	1.41 (1.05–1.90)	0.023	1.95 (0.95–4.02)	0.066
Age, <65 years (65 pairs)	NE		1.34 (0.82–2.18)	0.242	1.30 (0.85–2.00)	0.226	NE	
Hypertension (194 pairs)	NE		0.96 (0.72–1.27)	0.775	1.07 (0.84–1.38)	0.582	1.54 (0.80–2.95)	0.189
Dyslipidemia (174 pairs)	1.57 (0.91–2.69)	0.100	1.51 (1.13–2.02)	0.005	1.44 (1.11–1.87)	0.006	2.33 (1.27–4.28)	0.005
Chronic kidney disease (84 pairs)	1.22 (0.66–2.27)	0.529	1.39 (0.95–2.04)	0.088	1.68 (1.16–2.44)	0.006	1.61 (0.76–3.42)	0.207
Mastectomy (89 pairs)	1.09 (0.50–2.36)	0.828	1.43 (0.94–2.17)	0.095	1.34 (0.91–1.98)	0.139	NE	
Antineoplastic chemotherapy (196 pairs)	1.71 (1.08–2.72)	0.021	1.41 (1.07–1.85)	0.014	1.43 (1.12–1.84)	0.005	1.37 (0.75–2.52)	0.306
Hemoglobin A1c, >7% (44 pairs)	NE		2.65 (1.47–4.77)	0.001	2.08 (1.23–3.51)	0.005	NE	
Albumin, <3.5 g/dL (74 pairs)	1.54 (0.90–2.61)	0.109	1.52 (0.98–2.36)	0.061	1.18 (0.80–1.75)	0.395	1.76 (0.81–3.79)	0.146

CI, confidence interval; ED, emergency department; HR: hazard ratio; ICU, intensive care unit; NE: not estimable due to low events.

**Table 6:** Subgroup analyses of adverse outcomes within five years of the index date in the zinc deficiency group versus zinc normal control group after propensity score matching.

Subgroup	All-Cause Mortality		ED Visits		Hospitalizations		ICU Admissions	
	HR (95% CI)	*p*	HR (95% CI)	*p*	HR (95% CI)	*p*	HR (95% CI)	*p*
Age, ≥65 years (143 pairs)	1.41 (0.83–2.39)	0.198	1.55 (1.13–2.12)	0.006	1.42 (1.06–1.90)	0.019	1.54 (0.82–2.91)	0.177
Age, <65 years (65 pairs)	1.40 (0.64–3.04)	0.399	1.25 (0.78–2.01)	0.352	1.30 (0.85–2.00)	0.226	NE	
Hypertension (194 pairs)	1.48 (0.71–3.08)	0.288	0.94 (0.73–1.21)	0.623	1.05 (0.83–1.32)	0.703	1.79 (1.01–3.19)	0.045
Dyslipidemia (174 pairs)	1.52 (0.92–2.50)	0.099	1.39 (1.06–1.84)	0.019	1.44 (1.11–1.87)	0.006	2.07 (1.21–3.54)	0.006
Chronic kidney disease (84 pairs)	1.29 (0.73–2.30)	0.379	1.33 (0.92–1.93)	0.131	1.63 (1.13–2.36)	0.008	1.37 (0.70–2.69)	0.358
Mastectomy (89 pairs)	1.12 (0.53–2.36)	0.768	1.37 (0.91–2.05)	0.129	1.34 (0.91–1.98)	0.139	NE	
Antineoplastic chemotherapy (196 pairs)	1.57 (1.02–2.41)	0.039	1.29 (0.99–1.67)	0.061	1.44 (1.12–1.85)	0.004	1.18 (0.68–2.05)	0.549
Hemoglobin A1c, >7% (44 pairs)	NE		2.09 (1.22–3.60)	0.006	2.00 (1.19–3.36)	0.008	NE	
Albumin, <3.5 g/dL (74 pairs)	1.47 (0.90–2.41)	0.119	1.44 (0.94–2.22)	0.096	1.15 (0.78–1.69)	0.485	1.69 (0.86–3.30)	0.123

CI, confidence interval; ED, emergency department; HR: hazard ratio; ICU, intensive care unit. NE: not estimable due to low events.

## Data Availability

The data that support the findings of this study were provided by TriNetX, LLC. These data are de-identified electronic health records (EHR) and are available via the TriNetX platform to researchers with a valid institutional agreement. Due to the proprietary nature of the database and the terms of the Data Use Agreement, the authors are not permitted to share the raw, patient-level data. Access can be requested directly through TriNetX at https://www.trinetx.com (accessed on 4 April 2026).

## Supplementary Materials

The following supporting information can be downloaded at: https://www.mdpi.com/article/10.3390/healthcare14091130/s1, Table S1: Study variable definitions, cohort-building logic, and TriNetX query codes. Table S2: STROBE/RECORD reporting checklist. Figures S1–S3: Kaplan–Meier event-free curves for (a) all-cause mortality, (b) emergency department visits, (c) hospitalizations, and (d) intensive care unit admissions within one year, three years, and five years after the index date. Reference [60] is cited in the Supplementary Table S2.

## Abbreviations

The following abbreviations are used in this manuscript:

CI	Confidence intervals
CPT	Current Procedural Terminology
HbA1c	Hemoglobin A1c
HR	Hazard ratio
ICD-10-CM	International Classification of Diseases, 10th Revision, Clinical Modification
ICU	Intensive care unit
IDF	International Diabetes Federation
LOINC	Logical Observation Identifiers Names and Codes
ZC	Normal zinc control
ZD	Zinc deficiency
ZH	High zinc
ZMRG	Zinc metabolism-related gene
ZNF207	Zinc finger protein 207
