# Supplementary material for: Association of Serum Zinc Status with 5-Year Clinical Outcomes in Women with Breast Cancer and Type 2 Diabetes: A Retrospective Cohort Study Using TriNetX
Source: Healthcare (Basel). 2026 Apr 23;14(9):1130. doi: 10.3390/healthcare14091130 (PMC13163481; doi:10.3390/healthcare14091130)
Supplement: Supplementary file 1 [file healthcare-14-01130-s001.zip › Supplementary Figure S2.pdf]

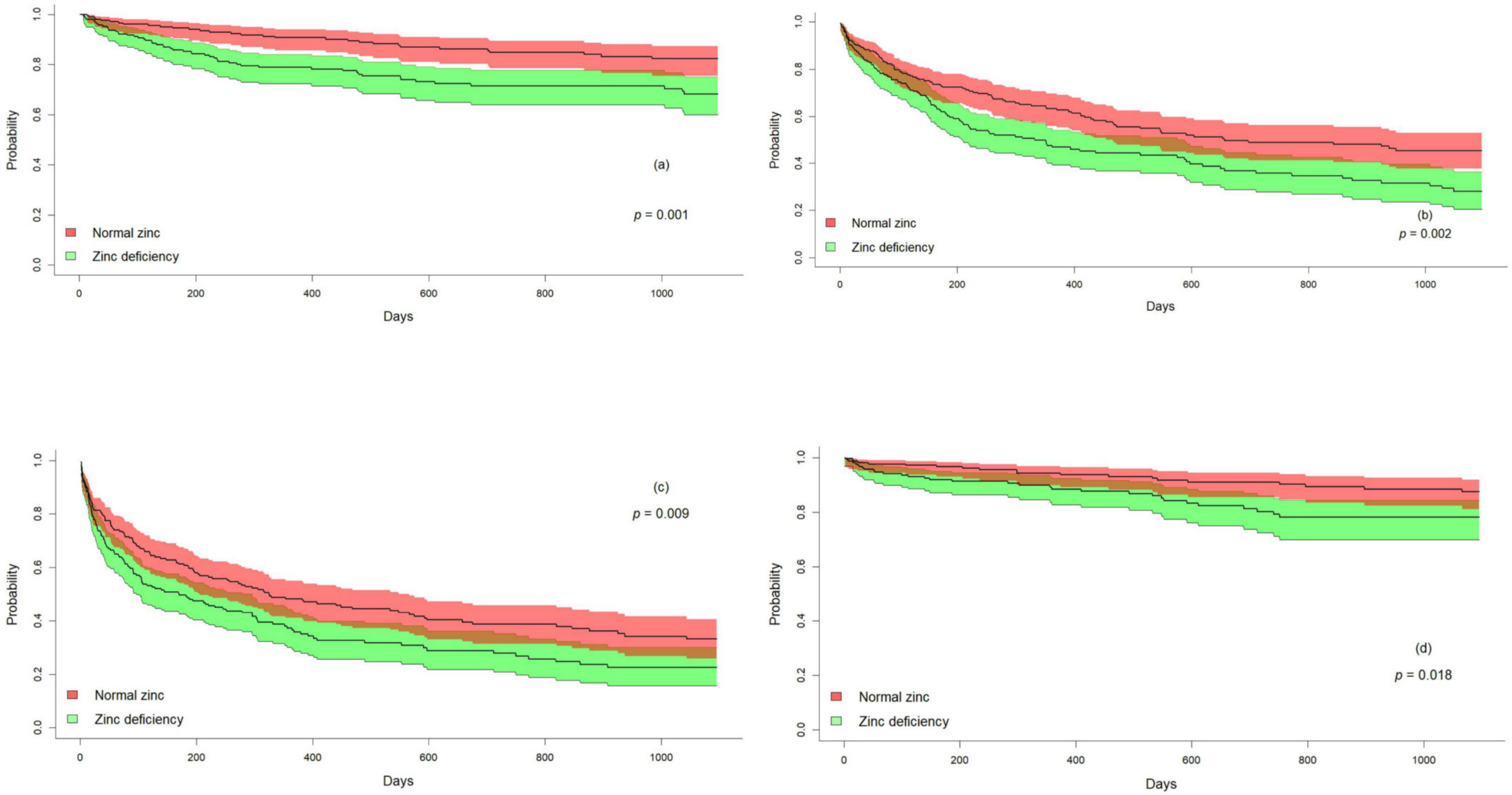

Supplementary Figure S2. Kaplan–Meier event-free curves for (a) all-cause mortality, (b) emergency department visits, (c) hospitalizations, and (d) intensive care unit admissions within three years after the index date
