# Supplementary material for: Association of Serum Zinc Status with 5-Year Clinical Outcomes in Women with Breast Cancer and Type 2 Diabetes: A Retrospective Cohort Study Using TriNetX
Source: Healthcare (Basel). 2026 Apr 23;14(9):1130. doi: 10.3390/healthcare14091130 (PMC13163481; doi:10.3390/healthcare14091130)
Supplement: Supplementary file 1 [file healthcare-14-01130-s001.zip › Supplementary Table S1.pdf]

Table S1. Definitions and TriNetX codes for study variables.

Category	Variable Name	TriNetX Code / Identifier
**Cohort Selection**		
	Malignant neoplasm of breast	UMLS:ICD10CM:C50
	Type 2 diabetes mellitus	UMLS:ICD10CM:E11
**Exposure (Zinc status)**		
	Zinc (Mass/volume) in serum or Plasma	UMLS:LNC:5763-8
	Zinc (mass/volume) in blood	UMLS:LNC:8245-3
**Outcomes**		
All-cause mortality	Deceased or ill-defined and unknown cause of mortality	UMLS:ICD10CM:R99
Emergency department visits	Emergency department services	UMLS:CPT:1013711
	Visit: Emergency	UMLS:HL7V3.0:VisitType:EMER
Hospitalizations	Visit: Inpatient encounter	UMLS:HL7V3.0:VisitType:IMP
	Visit: Short stay	UMLS:HL7V3.0:VisitType:SS
	Visit: Inpatient non-acute	UMLS:HL7V3.0:VisitType:NONAC

	Visit: Inpatient acute	UMLS:HL7V3.0:VisitType:ACUTE
Intensive care unit admissions	Critical care services	UMLS:CPT:1013729
**Covariates (Propensity Score Matching)**		
Demographics		
	Age at Index	AI
	Sex	Female (F)
	Race / Ethnicity	White (2106-3), Black or African American (2054-5), Not Hispanic or Latino (2186-5), Unknown (UN)
Comorbidities		
	Essential hypertension	ICD-10-CM: I10
	Cerebral infarction	ICD-10-CM: I63
	Malnutrition	ICD-10-CM: E40-E46
	Hyperlipidemia, unspecified	ICD-10-CM: E78.5
	Chronic kidney disease	ICD-10-CM: N18
Treatments & Procedures		
	Mastectomy Procedures	1015054

	Radiotherapy	1287742003
	Chemotherapy	1002
Laboratory & Medication		
	Hemoglobin A1c	43396009
	Albumin	1011249
	Tamoxifen	10324

CPT, Current Procedural Terminology; ICD-10-CM, International Classification of Diseases, 10th Revision, Clinical Modification; UMLS, Unified Medical Language System.
